# Sodium nitrite exerts an antihypertensive effect and improves endothelial function through activation of eNOS in the SHR

**DOI:** 10.1038/srep33048

**Published:** 2016-09-12

**Authors:** Wei Chih Ling, Dharmani Devi Murugan, Yeh Siang Lau, Paul M. Vanhoutte, Mohd Rais Mustafa

**Affiliations:** 1 Department of Pharmacology, Faculty of Medicine, University of Malaya, 50603 Kuala Lumpur, Malaysia; 2 State Key Laboratory for Pharmaceutical Biotechnology, Department of Pharmacology and Pharmacy and University of Hong Kong, Hong Kong, China

## Abstract

Sodium nitrite (NaNO_2_) induces relaxation in isolated arteries partly through an endothelium-dependent mechanism involving NO-eNOS-sGC-cGMP pathway. The present study was designed to investigate the effect of chronic NaNO_2_ administration on arterial systolic blood pressure (SBP) and vascular function in hypertensive rats. NaNO_2_ (150 mg L−1) was given in drinking water for four weeks to spontaneously (SHR) and Nω-Nitro-L-arginine methyl ester hydrochloride (L-NAME) treated hypertensive SD rats. Arterial SBP and vascular function in isolated aortae were studied. Total plasma nitrate/nitrite and vascular cyclic guanosine monophosphate (cGMP) levels were measured using commercially available assay kits. Vascular nitric oxide (NO) levels were evaluated by DAF-FM fluorescence while the proteins involved in endothelial nitric oxide synthase (eNOS) activation was determined by Western blotting. NaNO_2_ treatment reduced SBP, improved the impaired endothelium-dependent relaxation, increased plasma total nitrate/nitrite level and vascular tissue NO and cGMP levels in SHR. Furthermore, increased presence of phosphorylated eNOS and Hsp-90 was observed in NaNO_2_-treated SHR. The beneficial effect of nitrite treatment was not observed in L-NAME treated hypertensive SD rats. The present study provides evidence that chronic treatment of genetically hypertensive rats with NaNO_2_ improves endothelium-dependent relaxation in addition to its antihypertensive effect, partly through mechanisms involving activation of eNOS.

Nitric oxide (NO) is an endothelium-derived relaxation factor which regulates multiple biological processes including the control of the vascular tone, cardiac and vascular remodeling, platelet aggregation and vascular smooth muscle cell proliferation^1^,^2^,^3^. The production of NO from L-arginine, oxygen and cofactors results mainly from the activity of nitric oxide synthase (NOS)^4^,^5^.

Molecular and enzymatic studies indicate that at least three distinct NOS isoforms exist in mammalian cells: endothelial (eNOS), neuronal (nNOS) and inducible (iNOS)^6^. In the endothelium, NO is formed mainly by eNOS^7^. Dynamic interactions of several inhibitory and stimulatory proteins in an isoform-specific manner influence the NOS enzymatic activity. One of these protein is heat shock protein, Hsp90, which plays an essential role in normal cellular homeostasis and protects cells from damage in response to stress^8^,^9^. Binding of Hsp-90 to eNOS stimulates its activity by enhancing the catalytic functions of the enzyme and maintaining the balance between eNOS-produced NO and superoxide anions (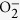)^8^,^10^,^11^.

NO is a relatively unstable molecule. It is rapidly oxidized into its relatively stable anions, namely nitrite (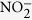) and nitrate (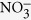)^12^. Nitrite has long been viewed as a diagnostic marker for NO production in biological systems and has been considered to be an inert NO product. However, several studies suggest that besides being a simple marker for NO formation, it can be reduced back to NO by several enzymes and such reduction is enhanced by hypoxia^13^,^14^,^15^. Hence, it remains questionable whether or not endogenous nitrite contributes to vasodilatation under normal physiological conditions.

Supplementation of sodium nitrite to genetically hypertensive animals causes a decrease in arterial blood pressure^16^,^17^. However, the exact mechanism leading to this response is yet to be elucidated. Previous work has shown that exogenous nitrite induces endothelium-dependent relaxation in isolated aortae of spontaneously hypertensive rats by activating the eNOS-NO-soluble guanylyl cyclase (sGC)-cyclic guanosine monophosphate (cGMP) pathway^18^. The present experiments were designed to investigate the effect of *in vivo* treatment with sodium nitrite on arterial blood pressure and endothelial dysfunction in two different model of hypertension and to examine the role of eNOS activation in the observed effects.

## Results

### Sodium nitrite decreased systolic blood pressure in SHR

**Figure 1:**
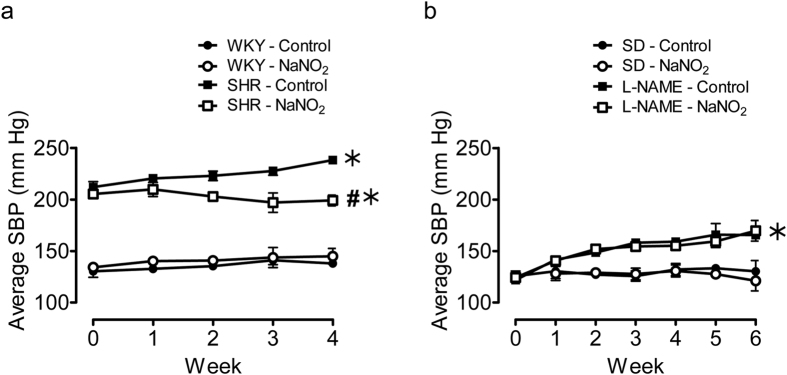
Sodium nitrite treatment for four weeks decreased average systolic blood pressure of SHR but not L-NAME hypertensive rats. Measurement of average systolic blood pressure by tail cuff method following four weeks of *in vivo* sodium nitrite treatment in SHR (**a**) and L-NAME treated hypertensive SD rats (**b**). Data are expressed as means ± SEM (n = 6–8). ^#^p ≤ 0.05 compared to respective control; *p ≤ 0.05 compared to normotensive control.

The basal systolic blood pressure of Spontaneously Hypertensive Rats (SHR) was significantly higher than that of Wistar Kyoto rats (WKY) (Fig. 1a). Sodium nitrite treatment for four weeks decreased this parameter significantly in SHR rats. The baseline systolic blood pressure of untreated (control) and Nω-Nitro-L-arginine methyl ester hydrochloride (L-NAME) treated Sprague Dawley (SD) rats was similar (Fig. 1b). An increase in systolic blood pressure was observed after the first week of L-NAME treatment, and this increase was continuous in all L-NAME treated animals, with or without sodium nitrite treatment. No significant changes in systolic blood pressure were observed following sodium nitrite treatment in WKY and normotensive SD rats. The chronic treatment with sodium nitrite had no significant effect on body weight (data not shown).

### Sodium nitrite improved endothelium-dependent relaxation in SHR aorta

**Figure 2:**
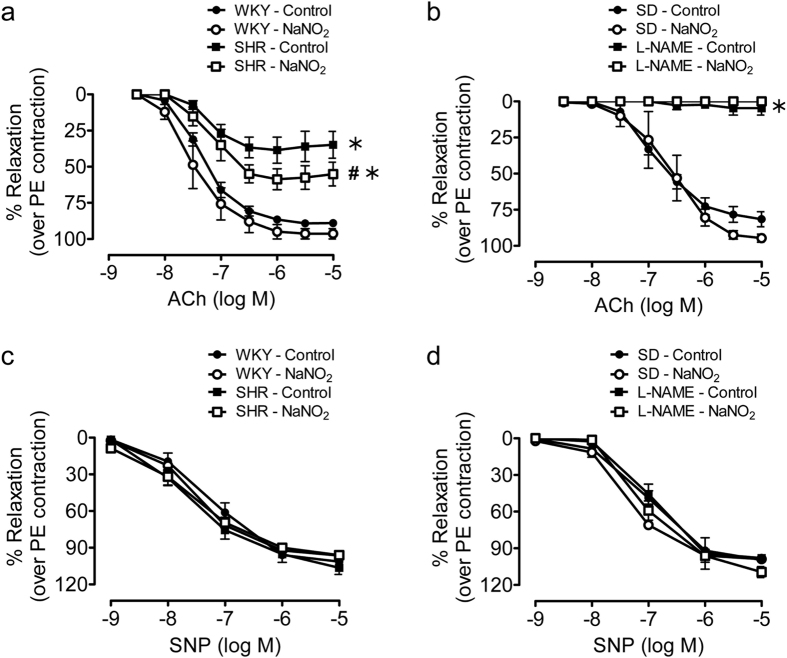
*In vivo* treatment of sodium nitrite for four weeks increased relaxation of SHR aortic rings to acetylcholine but not L-NAME treated hypertensive rats aortic rings. Relaxation to increasing concentrations of acetylcholine (ACh) in aortic rings of WKY and SHR (**a**) or control and L-NAME treated hypertensive SD rats (**b**), with or without the treatment of sodium nitrite. (**c**,**d**) Relaxation to increasing concentrations of sodium nitroprusside (SNP). Data are expressed as means ± SEM (n = 6–8). ^#^p ≤ 0.05 compared to respective control; *p ≤ 0.05 compared to normotensive control.

The relaxation to acetylcholine were decreased in aortic rings of SHR and L-NAME-treated hypertensive rats, compared to their respective controls. *In vivo* treatment with sodium nitrite improved the impaired acetylcholine-induced relaxation in SHR aortae (Fig. 2a), but this effect was not observed in rings of L-NAME-treated hypertensive rats (Fig. 2b). The relaxation to the exogenous NO donor, sodium nitroprussides was similar in aortae of the different experimental groups (Fig. 2c,d).

### Sodium nitrite increased the total NO and cGMP level in SHR aorta

**Figure 3:**
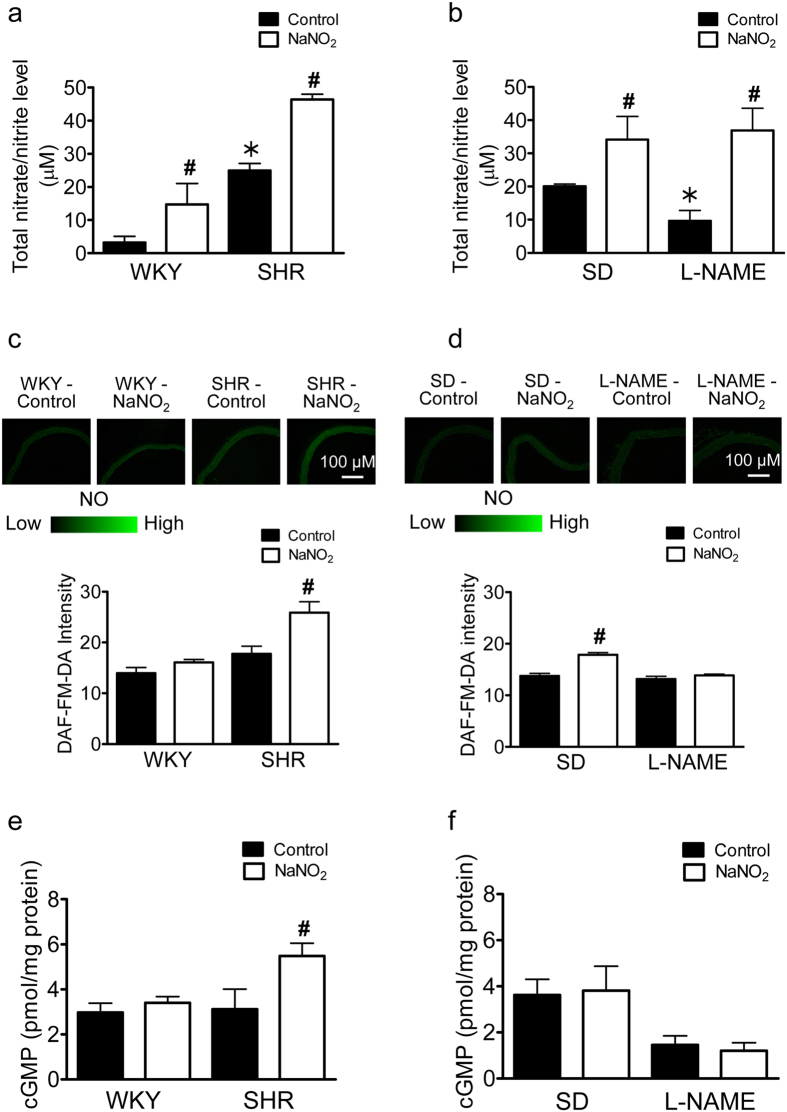
Sodium nitrite treatment for four weeks increased plasma total nitrite/nitrate level, NO level and total cGMP level in the aorta of SHR but not L-NAME treated hypertensive SD rats. The plasma total nitrite/nitrate level of WKY and SHR (**a**) or of control and L-NAME treated hypertensive SD rats (**b**), with or without treatment with sodium nitrite measured by commercially available colorimetric assay kit. NO levels in the aortic tissues of WKY and SHR (**c**) or of control and L-NAME treated hypertensive SD rats (**d**), with or without the treatment with sodium nitrite measured by DAF-FM fluorescence. Aortic cGMP levels in rings of WKY and SHR (**e**) or of control and L-NAME treated hypertensive SD rats (**f**), with or without treatment with sodium nitrite measured by ELISA kits. Data are expressed as means ± SEM (n = 6–8). ^#^p ≤ 0.05 compared to respective control; *p ≤ 0.05 compared to normotensive control.

SHR control animals has significantly higher plasma levels of total nitrate/nitrite compared to WKY controls (Fig. 3a), while in L-NAME treated hypertensive animals a significant decrease in the plasma total nitrate/nitrite level was observed compared to SD controls (Fig. 3b). All animal group treated with sodium nitrite showed a significant increase in plasma total nitrate/nitrite level. Measurement of *in situ* NO production by 4-amino-5-Methylamino-2’, 7-Difluorofluorescein Diacetate (DAF-FM) fluorescence staining revealed that aortic rings of sodium nitrite treated SHR exhibited a significant increase in NO level compared to controls (Fig. 3c). This increase was not observed in preparations of L-NAME treated SD hypertensive rats receiving a similar sodium nitrite treatment (Fig. 3d). The increase in NO level in aortae of SHR treated with sodium nitrite was accompanied by an increase in total cGMP level (Fig. 3e) but no changes were seen in preparations of L-NAME treated SD rats (Fig. 3f).

### Sodium nitrite increased the expression of phosphorylated eNOS and Hsp-90 in SHR

**Figure 4:**
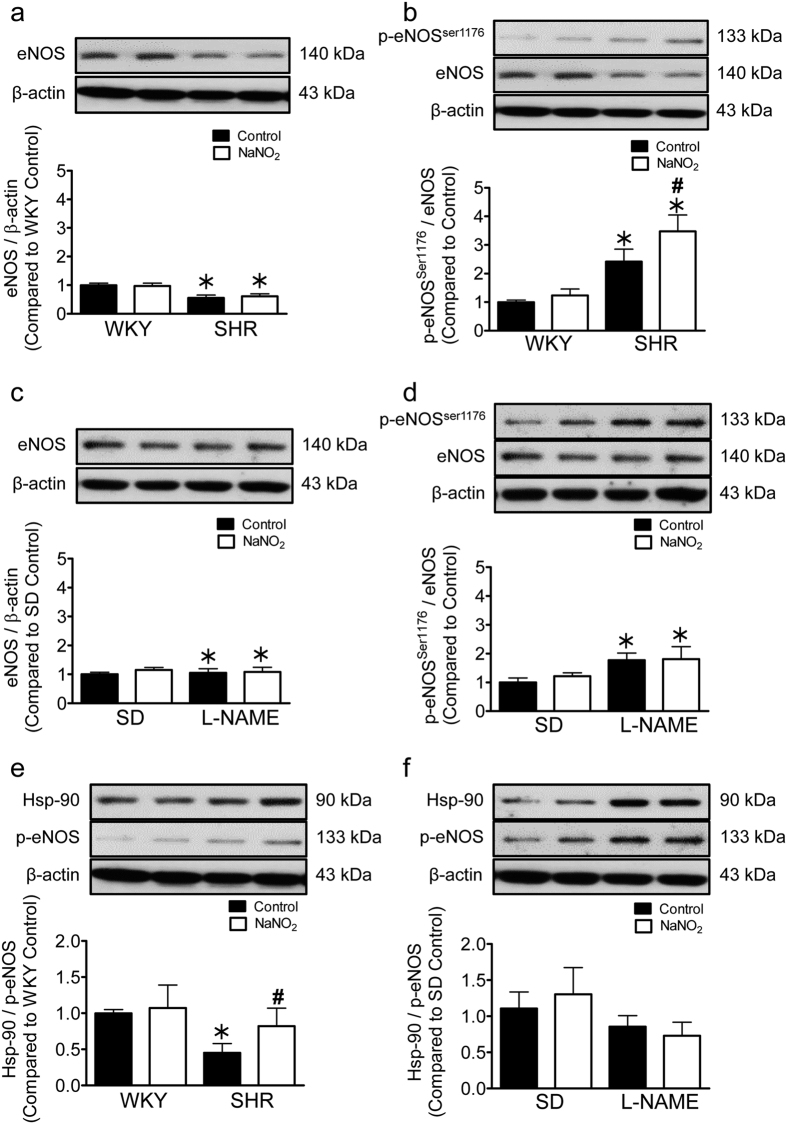
Sodium nitrite treatment for four weeks increased the level of phosphorylated Enos (p-eNOS) and Hsp-90 to p-eNOS ratio in SHR aorta but not in L-NAME treated hypertensive rats. Presence of total eNOS, p-eNOS and Hsp-90 in aortic tissues of WKY and SHR (**a**,**b**,**e**) or of control and L-NAME treated hypertensive SD rats (**c**,**d**,**f**), with or without the treatment with sodium nitrite detected by Western blotting. The upper panels shows a representative Western blots and the bottom panel shows the ratio of protein to β–actin (**a**,**c**), eNOS (**b**,**d**) or p-eNOS (**e**,**f**). Data are expressed as means ± SEM (n = 5–8). ^#^p ≤ 0.05 compared to respective control; *p ≤ 0.05 compared to normotensive control.

Western blotting revealed that the presence of total eNOS was decreased in the aortae of SHR compared to WKY preparations and was not affected by sodium nitrite treatment (Fig. 4a). The aortae of SHR animals exhibited a significantly higher level of phosphorylated eNOS compared to those of WKY and this level was further increased after four weeks of sodium nitrite treatment (Fig. 4b). The presence of total eNOS were not altered in aortae from L-NAME treated hypertensive SD rats, and was not affected by the sodium nitrite treatment compared to controls (Fig. 4c). The ratio of phosphorylated eNOS to total eNOS was significantly higher in L-NAME treated rats but was not significantly altered by the additional sodium nitrite treatment (Fig. 4d). The ratio of Hsp-90 to phosphorylated eNOS in SHR aortic tissues was significantly lower compared to WKY preparations; this ratio was increased in aortae of SHR but not in L-NAME hypertensive SD rats animals treated with sodium nitrite (Fig. 4e,f).

## Discussion

The present study demonstrates that *in vivo* treatment with sodium nitrite effectively reduces arterial blood pressure in genetically hypertensive SHR rats, and improves endothelium-dependent relaxations in their aortae, together with increasing the phosphorylation of eNOS and Hsp-90 expression. These effects of *in vivo* sodium nitrite administration were not observed in WKY rats, which, although they are derived from genetically different sublines^19^, are the conventional normotensive controls used for comparison with the SHR^20^,^21^,^22^,^23^. Likewise, the beneficial effect of *in vivo* sodium nitrite treatment is absent in animals made hypertensive by inhibition of the NOS enzyme by L-NAME.

Earlier work had demonstrated that sodium nitrite acutely elicited endothelium-dependent relaxation in isolated SHR aortic rings through activation of eNOS^18^. The present findings show that four weeks of sodium nitrite treatment successfully decreased the arterial blood pressure and improved endothelium-dependent relaxations in the SHR. This decreased in arterial blood pressure and improvement in endothelial function were not observed in L-NAME treated hypertensive animals, which may be explained by the persistent inhibition of eNOS. However, the lack of effect in decreasing blood pressure by sodium nitrite treatment in the latter animals may also be due to the fact that blood pressure of the L-NAME treated SD rats in the present study did not reach the same level as obtained by others^24^,^25^ and is not comparable in amplitude as that observed in the SHR used. The beneficial effect of the chronic treatment with sodium nitrite is not explained by an enhanced sensitivity of SHR vascular smooth muscle to NO, as relaxations to the exogenous NO donor, sodium nitroprusside were not affected implying that the improvement by sodium nitrite is due to changes in bioavailability of endothelium-derived NO rather than a direct effect on the underlying vascular smooth muscle cells.

Increasing the NO bioavailability in the vascular system has been viewed as a major therapeutic approach in hypertension^26^,^27^,^28^. Carlstrom and colleagues showed the existence of a cross-talk between the nitrate-nitrite-NO pathway and the classical L-Arginine-NOS dependent system in the control of NO homeostasis^26^. Sodium nitrite exhibits antihypertensive properties in SHR, two-kidney, one-clip (2K1C) and salt-induced hypertensive animal models^17^,^29^,^30^,^31^. However, to judge from the results obtained in normal SD rats and C57BL/6NCrl mice, chronic supplementation with nitrate is associated with vascular eNOS downregulation in rodents suggesting that the response to dietary nitrate or nitrite depends on the basal eNOS activity^26^. Reduction of arterial blood pressure by dietary nitrate or nitrite may be more pronounced in young and healthy subjects whose eNOS is operating at maximal capacity as stimulation of the nitrate-nitrite pathway may downregulates normal vascular eNOS activity. However, in individuals with compromised eNOS activity, dietary nitrate or nitrite supplementation could increase NO bioavailability, which may be the situation in the present study. In line with this interpretation, nitrate treatment lowers arterial blood pressure and prevents adverse cardiovascular outcomes in salt-induced hypertension^31^. Although the present study demonstrates the important role of endothelium derived NO (EDNO) bioavailability for the action of sodium nitrite, other possible mechanisms contributing to its antihypertensive action cannot be excluded. Thus, in 2K1C hypertensive rats sodium nitrite exerts an indirect antioxidant effect, most probably due to downregulation of NADPH oxidase activity^30^. In addition, the antihypertensive effect of orally administered nitrate or nitrite may also be due to increased formation of S-nitrosothiols under acidic condition in the stomach^32^.

The present study confirms that untreated SHR rats exhibit a higher total nitrate/nitrite plasma level compared to the WKY controls, which is in agreement with several early studies^20^,^33^,^34^. An increase in iNOS activity^33^, release of NO from protein-bound dinitrosyl nonheme iron complexes (DNIC) in the vasculature and NOS-independent release of NO^20^ may explain the elevated total nitrate/nitrite level in the SHR. On the other hand, the total plasma nitrate/nitrite level was decreased in the control group of L-NAME treated hypertensive animals, which was to be expected as eNOS is inhibited and can no longer effectively produce NO under these conditions. All animals treated with sodium nitrite exhibited an increased total plasma nitrate/nitrite level, indicating that sodium nitrite was delivered effectively in the drinking water^30^,^35^.

The beneficial antihypertensive effects of *in vivo* sodium nitrite in SHR is associated with increased production of NO, as shown by the DAF-FM fluorescence assay done. Previous studies indicated the existence of a pathway that converts 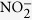 to NO^20^,^36^,^37^. This conversion also occurs after oral ingestion of nitrite^38^. NO acts as a paracrine regulator of the underlying vascular smooth muscle tone by activating sGC to produce cGMP which then induces relaxation^39^. In the current study, the changes in plasma total nitrate/nitrite level and DAF-FM fluorescence signal are paralleled by increases in aortic tissue cGMP level. Indeed, the aortae of SHR treated with sodium nitrite exhibited increased cGMP levels compared to controls. On the other hand, although there is an increase in plasma total nitrate/nitrite level in L-NAME treated hypertensive SD rats treated with sodium nitrite, there is no further increased in DA-FM signal and cGMP level in the aortae of these animals. The absence of increased DAF-FM signal and cGMP level in the aortae from L-NAME treated animals, compared to that observed in aortae of SHR following *in vivo* treatment with sodium nitrite, suggests that functional eNOS is required in order for the nitrite supplement to exert its beneficial effects in the hypertensive animals. Indeed, the absence of relaxations to acetylcholine in aortae of L-NAME treated SD rats demonstrates the non-functionality of eNOS under such experimental conditions.

Several factors can lead to reduction of NO synthesis by eNOS including changes in mRNA expression resulting in decreased protein presence, post-transductional modifications, interactions with endogenous modulators or suboptimal concentrations of substrate and cofactors^40^. In line with previous findings^41^,^42^, the present study shows that the basal presence of eNOS in SHR arteries is lesser than that in those of the WKY. However, there was a higher level of phosphorylated eNOS in the former. Sodium nitrite treatment for four weeks further increased the expression of phosphorylated eNOS in SHR preparations, suggesting that the antihypertensive effect of sodium nitrite involved activation of eNOS, which eventually leads to the production of NO, as demonstrated by the increased in DAF-FM signal and cGMP level in the treated SHR aortic tissues. On the other hand, the same treatment with sodium nitrite in L-NAME induced hypertensive animals did not cause an increase in phosphorylated eNOS, an observation which further supports that a functional eNOS is required for sodium nitrite to exert its antihypertensive effect.

Experiments on human umbilical vein endothelial cells (HUVECs) and isolated rat aorta have demonstrated that eNOS activity is enhanced by the complex formation between eNOS and Hsp-90, leading to increased production of NO^43^. The current measurements of Hsp-90 protein expression demonstrate that four weeks of sodium nitrite treatment increased the Hsp-90/phosphorylated eNOS ratio in vascular tissues from SHR. This may imply that sodium nitrite exerts its antihypertensive effect at least in part by facilitating the interaction of Hsp-90 with eNOS, leading to the improved phosphorylation hence activation of this enzyme.

In summary, the present study demonstrates that in genetically hypertensive rats chronic treatment with sodium nitrite reduces arterial blood pressure and improves endothelium-dependent relaxations most probably through the activation of eNOS, resulting at least in part from a facilitated interaction between Hsp-90 and the enzyme, which leads to an increased production of NO and resulting augmented cGMP levels. Thus, these finding indicate that sodium nitrite may potentially be useful for the treatment of hypertension by improving endothelial function and correcting vascular NO deficiency.

## Methods

### Animals

The experimental procedures were approved by the Animal Care and Ethics Committee of the University of Malaya. Twelve to 14 weeks old male Wistar Kyoto rats (WKY), Spontaneously Hypertensive Rats (SHR) and Sprague Dawley (SD) rats were purchased from Monash University (Sunway Campus, Malaysia). The animals were housed in cages at a controlled temperature (22–25 °C) and lighting condition (12-hours light/dark cycle) with free access to standard chow and water. All animal studies were in compliance with the ARRIVE guidelines for experiments involving animals^44^.

WKY and SHR were randomly divided into four experimental groups: WKY Control (normal drinking water), WKY + nitrite (sodium nitrite, 150 mg L^−1^, for four weeks), SHR Control (normal drinking water), and SHR + nitrite. SD rats were also randomly divided into four experimental groups: SD Control (normal drinking water), SD + nitrite, L-NAME controls (L-NAME, 60 mg kg^−1^ day^−1^) and L-NAME + nitrite. The L-NAME treated animals were given the NOS inhibitor for two weeks prior to and during the sodium nitrite treatment. L-NAME and sodium nitrite were dissolved in normal drinking water.

### Arterial systolic blood pressure

The average systolic blood pressure of the animals was measured by the tail-cuff method (NIBP monitoring system, IITC Inc, Woodland Hills, CA, USA) before all treatments, and every subsequent week after the treatment started. Briefly, all animals were trained to the restraint condition before measurement. They were immobilized in a pre-warmed chamber (28–30 °C) for at least 30 minutes before each blood pressure measurement was carried out. At least six to seven successive measurements were recorded and the average values of these readings are reported^45^.

### Vascular function

At the end of treatment, the animals were sacrificed and the thoracic aorta was isolated and placed in modified Krebs physiological salt solution [control solution, in mM: sodium chloride (NaCl) 118.93, sodium bicarbonate (NaHCO_3_) 25.00, magnesium sulphate (MgSO_4_) 1.18, potassium chloride (KCl) 4.69, potassium dihydrogen phosphate (KH_2_PO_4_) 1.03, Glucose 11.10, calcium chloride (CaCl_2_) 2.38]. The blood vessels were cut into segments of 3–4 mm length; some of the segments were snap frozen in liquid nitrogen and stored in −80 °C for later processing. Aortic segments for isometric tension measurement were suspended in organ chamber containing 5 mL of control solution (37 °C), consistently aerated with 95% oxygen and 5% carbon dioxide. The rings were connected to a force transducer (Grass Instrument Co, Quincy, MA, USA). Changes in isometric tension were recorded using a PowerLab recording system (AD Instruments, Sydney, Australia). After an equilibration for 60 minutes at a resting tension of 1.0 g, the viability of the rings was tested by the addition of 60 mM KCl until a stable contraction was achieved. The rings were then washed three times with control solution, followed by addition of phenylephrine (300 nM–1 µM) to induce a stable contraction. Cumulative concentration-responses to the endothelium-dependent relaxation agonist acetylcholine (ACh, 3 nM–10 μM) and to the endothelium-independent vasodilator sodium nitroprusside (1 nM–10 μM) were then obtained. Relaxations are expressed as percentage of the precontraction level obtained with phenylephrine.

### Total nitrate/nitrite levels

Blood samples were collected at the end of the experiment from all rats by cardiac puncture; the total nitrate/nitrite concentration was determined in the plasma using a colorimetric assay kit (Cayman Chemical Company, Ann Arbor, MI, USA). The absorbance was measured using a plate reader (Tecan, Männedorf, Schweiz) at 540 nm. The results are expressed in μM.

### Nitric oxide (NO) production

*In situ* NO production was determined using DAF-FM diacetate (Invitrogen, Carlsbad, CA, USA) (Altaany *et al*.^46^). Isolated aortae from the animals were embedded in OCT compound (Sakura Finetik, AJ Alphen aan den Rijn, Netherlands) until frozen. The frozen aortic segments were cut into 5 μm thick sections using a cryostat (Thermo Fisher Scientific, Waltham, MA, USA). The sections were then incubated for 30 minutes in normal physiological saline solution [NPSS (mM): NaCl 140, KCl 5, CaCl_2_ 1, MgCl_2_ 1, glucose 10 and HEPES 5] containing 5 μM DAF-FM diacetate at 37 °C for 15 minutes. The images were then viewed with a fluorescence microscope (Leica Microsystems, Wetzlar, Germany) with excitation at 495 nM and emission at 515 nM, and the fluorescence intensity was measured using the Leica LAS-AF software version2.6.0.

### Tissue levels of cGMP

Aortae were homogenized in 0.1 M hydrochloric acid and centrifuged at 15,000 g for 15 minutes at room temperature. The supernatant was used for the measurement of total cGMP using a commercially available assay kit (Direct cGMP ELISA Kit; Enzo Life Sciences Inc., Farmingdale, NY, USA). The results are expressed in pmol/mg protein.

### Western blotting

The aortae were homogenized in ice-cold 1X RIPA buffer (leupeptin 1 μg mL^−1^, aprotonin 5 μg mL^−1^, PMSF 100 μg mL^−1^, sodium orthovanadate 1 mM, EGTA 1 mM, EDTA 1 mM, NaF 1 mM, andβ-glycerolphosphate 2 mg mL^−1^). The lysate were centrifuged at 15,000 g for 30 minutes at 4 °C and the supernatant was used for Western blotting. Modified Lowry assay (Bio-Rad Laboratories, Hercules, CA, USA) was used to determine the protein concentrations of the supernatant. Twenty micrograms of total tissue protein for each sample were separated in 7.5% or 12% sodium dodecyl sulphate (SDS)-polyacrylamide gel and transferred to an immobilon-P polyvinylidene difluoride membrane (Millipore, Billerica, MA, USA) at 110 V for 90–120 minutes. The blots were then blocked for non-specific binding by incubation with 3% bovine serum albumin (BSA) in Tris-buffered saline containing 0.2% Tween-20 (TBS-T) at room temperature under gentle shaking. Later on, the blots were incubated with either primary antibodies against eNOS (1;500, BD Transduction Laboratory, Oxford, UK), p-eNOS^ser1176^ (1:1000, Abcam, Cambridge, UK) and Hsp-90 (1:1000, Cell Signalling Technology, Danvers, MA, USA) overnight at 4 °C. Then, the membranes were washed three times with TBS-T, followed by incubation with respective secondary antibodies conjugated to horseradish peroxidase at room temperature for two hours. The blots were developed with Amersham™ ECL prime Western Blotting detection reagent (Amersham, Bukinghamshire, UK) using X-ray film. Densitometric analysis of the images was then performed with Quantity One 1D analysis software (Bio-Rad Laboratories, Hercules, CA, USA). Respective protein expression levels were normalized to the housekeeping protein, β-actin.

### Drugs and chemicals

Phenylephrine, acetylcholine chloride, sodium nitrite, sodium nitroprusside, L-NAME and Tween-20 were purchased from Sigma Chemicals (St Louis, MO, USA). NaCl was purchased from Calbiochem^®^ Merck (Darmstadt, Germany). MgSO_4_, KCl, KH_2_PO_2_, glucose and CaCl_2_ were purchased from BDH Laboratory Supplies (Poole, UK). Bovine serum albumin (BSA) was purchased from Santa Cruz (Dallas, Texas, USA). All compounds were dissolved in deionized water. The concentrations stated are expressed as final molar concentrations in the buffer. At the concentrations used, the vehicles had no significant effect on vascular responses (data not shown).

### Data analysis

All results are presented as means ± standard error of mean (SEM) for the number of rats (n) in each group. GraphPad Prism 5 (GraphPad Software, La Jolla, CA, USA) was used to analyze the concentration-response curves by non-linear regression fitting. Student’s t-test (comparison for two groups) and one-way ANOVA followed by Bonferroni’s multiple comparison tests (for more than two groups) were performed using the same statistical software. A P value of less than 0.05 was considered to indicate statistically significant differences.

## Additional Information

**How to cite this article**: Ling, W. C. *et al*. Sodium nitrite exerts an antihypertensive effect and improves endothelial function through activation of eNOS in the SHR. *Sci. Rep.* **6**, 33048; doi: 10.1038/srep33048 (2016).
